# Physiological Effects of Oxidative Stress Caused by Saxitoxin in the Nematode *Caenorhabditis elegans*

**DOI:** 10.3390/md21100544

**Published:** 2023-10-19

**Authors:** Haiyan Wu, Balakrishnan Prithiviraj, Zhijun Tan

**Affiliations:** 1 Key Laboratory of Testing and Evaluation for Aquatic Product Safety and Quality, Ministry of Agriculture and Rural Affairs, Yellow Sea Fisheries Research Institute, Chinese Academy of Fishery Sciences, Qingdao 266071, China; wuhy@ysfri.ac.cn; 2 Department of Plant, Food and Environmental Sciences, Faculty of Agriculture, Dalhousie University, Nova Scotia, NS B2N5E3, Canada; bprithiviraj@dal.ca; 3 State Key Laboratory of Mariculture Biobreeding and Sustainable Goods, Yellow Sea Fisheries Research Institute, Chinese Academy of Fishery Sciences, Qingdao 266071, China

**Keywords:** *Caenorhabditis elegans*, saxitoxin, oxidative stress, reproductivity, ATP

## Abstract

Saxitoxin (STX) causes high toxicity by blocking voltage-gated sodium channels, and it poses a major threat to marine ecosystems and human health worldwide. Our work evaluated the neurotoxicity and chronic toxicology of STX to *Caenorhabditis elegans* by an analysis of lifespan, brood size, growth ability, reactive oxygen species (ROS) and adenosine triphosphate (ATP) levels, and the overexpression of green fluorescent protein (GFP). After exposure to a series of concentrations of STX for 24 h, worms showed paralysis symptoms and fully recovered within 6 h; less than 5% of worms died at the highest concentration of 1000 ng/mL for first larval stage (L1) worms and 10,000 ng/mL for fourth larval stage (L4) worms. Declines in lifespan, productivity, and body size of *C. elegans* were observed under the stress of 1, 10, and 100 ng/mL STX, and the lifespan was shorter than that in controls. With STX exposure, the productivity declined by 32–49%; the body size, including body length and body area, declined by 13–18% and 25–27%, respectively. The levels of ROS exhibited a gradual increase over time, accompanied by a positive concentration effect of STX resulting in 1.14–1.86 times higher levels compared to the control group in L4 worms. Conversely, no statistically significant differences were observed between L1 worms. Finally, after exposure to STX for 48 h, ATP levels and GFP expression in *C. elegans* showed a significant dose-dependent increase. Our study reports the first evidence that STX is not lethal but imposes substantial oxidative stress on *C. elegans*, with a dose-responsive relationship. Our results indicated that *C. elegans* is an ideal model to further study the mechanisms underlying the fitness of organisms under the stress caused by paralytic shellfish toxins including STX.

## 1. Introduction 

Paralytic shellfish toxins (PSTs) comprise more than 57 molecules with a tetrahydropurine structure, and saxitoxin (STX) is the major component of PSTs. PSTs are produced primarily by bloom-forming algal species in both marine and freshwater ecosystems, particularly dinoflagellates belonging to the genera *Alexandrium*, *Gymnodinium*, *Pyrodinium*, and cyanobacteria [1,2]. PSTs pose major public health threats through paralytic shellfish poisoning in consumers, thus, causing symptoms including a tickling sensation or numbness around the lips, numbness of the extremities, gastrointestinal problems, and fatal respiratory paralysis [2]. Beyond humans, PSTs affects marine organisms, such as zooplankton [3,4,5,6], shellfish [7,8,9,10,11,12], finfish [13,14,15,16], crustaceans [17,18], and even marine mammals [19,20], through either direct exposure or dietary uptake. PSTs cause major negative effects on animal health, including physiological activity [6,11], reproductive development [9,12] and growth ability [21,22], and can even lead to mass mortality [14,15,23] and decreases in the biomass and yield of aquatic systems [2,9,15,19,24,25]. 

To our knowledge, PSTs affect organisms primarily through two mechanisms: neurotoxicity and oxidative stress. The well-known mechanism of neurotoxicity of STX is based on binding to voltage-gated sodium channels [26,27]. Acute toxicity not only inhibits physiological activity but also causes substantial mortality in contaminated organisms [28]. Generally, acute toxicity is caused by neurotoxicity, mainly at high doses of PSTs, particularly during red tides caused by dinoflagellates [14,15]. In natural seawater, the dose of PSTs is insufficient to directly kill marine organisms. These PSTs consequently accumulate in other marine organisms along the food chain, and are metabolized to low- or non-toxic metabolites [29,30,31]. 

The accumulation and metabolism of PSTs cause the generation of excessive ROS in marine organisms, and subsequently perturb the redox balance and cause oxidative stress in contaminated organisms. To date, oxidative stress due to PSTs has been reported in zooplankton [6,32], shellfish [11,33,34,35], finfish [22,36,37,38] and marine mammals [39,40]. Similar to other toxins, PSTs lead to ROS attacks on lipids, proteins, and DNA; damage to the structures of cells, tissues and organs; inhibition of the functions of physiological, growth and reproductivity; and, finally, a decrease in marine biomass. 

Despite many years of investigation, numerous questions surround the mechanisms of oxidative stress caused by PSTs, particularly regarding STX. Prior experiments have been performed in zooplankton, shellfish, finfish, mammals, and even cell lines, all of which have cell membranes with voltage-gated sodium channels. As described in previous studies [41,42], STX blocking channels not only alters the influx of sodium ions into cells but also has adverse effects on physiological function, similar to responses to oxidative stress. Consequently, experiments with organisms that have Na^+^ channels are not able to accurately assess oxidative stress caused by STX due to possible neurotoxic effects. Therefore, more work is needed on the oxidative stress caused by PSTs to provide a clearer explanation for the marine biomass decline in natural seawater. 

*Caenorhabditis elegans* is used as the test animal primarily because this worm has no voltage-gated sodium channels in its cell membranes [43]; therefore, PSTs do not cause neurotoxicity but instead cause oxidative stress in *C. elegans*. *C. elegans* has many advantages as a model in biological research, such as its small size, rapid generation time, and consistent brood size [44,45]. Importantly, the *C. elegans* nervous system has been mapped, and *C. elegans* was the first multicellular animal for which the genome sequence was completed [46]. In this study, we used an alternative approach based on the model organism *C. elegans* to study the oxidative stress caused by PSTs. We exposed *C. elegans* to solutions of STX, the basic component of PSTs. Several different endpoints of *C. elegans*, including lethality, lifespan, brood size, body length, body size, and reactive oxygen species (ROS) and adenosine triphosphate (ATP) levels, were tested. We also used green fluorescent protein (GFP) markers for specific neuronal populations to assess the response of protein expression under the oxidative stress caused by STX.

## 2. Results

### 2.1. STX Caused no Lethal Paralytic Reaction in C. elegans

First larval stage (L1) and fourth larval stage (L4) worms were used to assess the toxicity of STX to *C. elegans*. *C. elegans* showed abnormal behaviors, such as body bends and slow head thrashes. Some worms were paralyzed completely and continued ankylosing, showing no response to touch. However, most worms had fully recovered and appeared normal within 6 h; less than 5% worms died in total, and these deaths were as a result of regular culturing losses that have no statistical significance.

**Figure 1:**
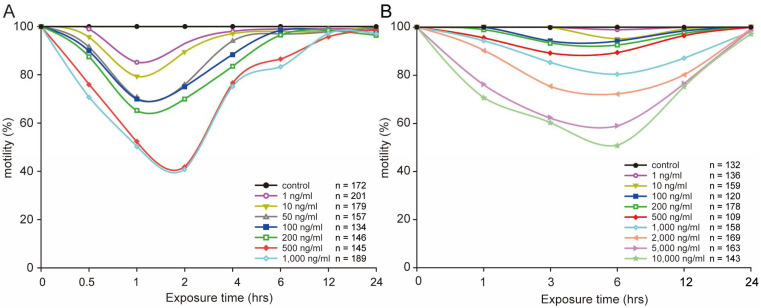
Influence of different concentrations of STX on L1 (**A**) and L4 (**B**) worms’ motility for 24 h. All data in the curve are mean values.

L1 worms were much more sensitive than L4 worms under the same STX treatment level, as shown in Figure 1. Approximately 59% of worms were completely paralyzed under the highest concentration of 1000 ng/mL in the L1 worms, as compared with 20% under the same concentration in the L4 worms. A serious negative effect on *C. elegans* was observed at the highest concentrations of 500, 1000 ng/mL for L1 worms and 10,000 ng/mL for L4 worms with the lowest motility below 50%. Generally, worms in the same stage showed dose–response trends under different concentrations of STX.

### 2.2. STX Induces the Production of ROS in Worms

**Figure 2:**
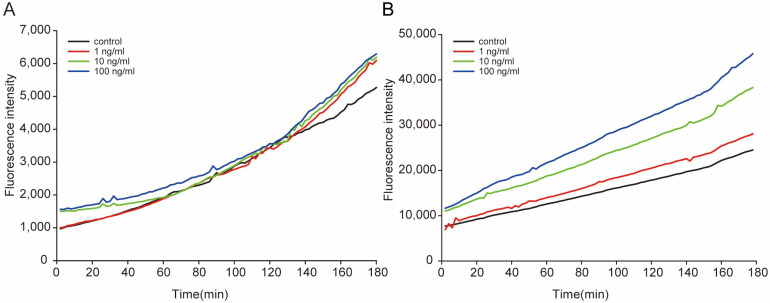
Dynamic variation in the general ROS in L1 (**A**) and L4 (**B**) worms after exposure to STX. The fluorescence intensity represents the relative concentrations of general ROS in worms, measured immediately after exposure.

We assessed the degree of oxidative stress in *C. elegans* under STX treatment by measuring the dynamic variation in general ROS produced by the worms. During the experiments, the ROS level increased with time, and the highest level was approximately four to six times greater than the lowest level in L1 worms (Figure 2A), and three to four times greater than that in L4 worms (Figure 2B). A positive concentration effect of STX on ROS production was observed in the L4 worms; the ROS levels at 1, 10, and 100 ng/mL were 1.14, 1.56, and 1.86 times higher than those of controls, respectively, at the end of the experiment. During the experiment, the ROS levels in L4 worms were much higher than those in L1 worms, mainly because of the body sizes of the worms.

### 2.3. STX Induced Body Size Decrease in C. elegans

**Figure 3:**
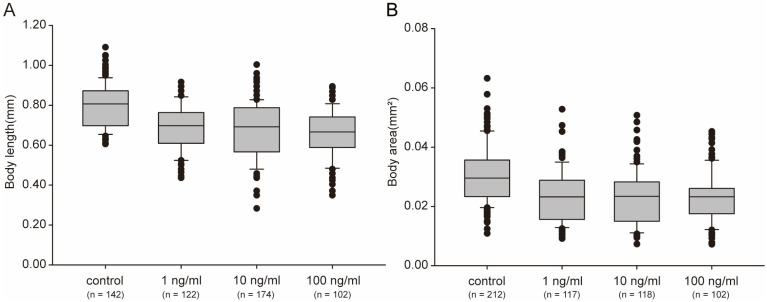
Body sizes, measured as length (**A**) and area (**B**), clearly decreased after treatment with STX for 48 h. Bars represent means ± SD, and different superscript letters indicate significant differences (*p* < 0.05). The horizontal lines represent the median, the hinges of the box plots represent 1st and the 3rd quartile, the whiskers are plotted at a distance of 1.5 times the inter-quartile range, the thick dots represent outliers and the thin dots represent the raw data.2.4. STX Shortens the Lifespan of *C. elegans*.

Body size directly contributes to the biomass of animals and is also closely associated with lifespan. In this study, worm growth was significantly impaired after STX exposure. The body length and body area of worms treated with STX were smaller than those in the control (*p* ˂ 0.05) (Figure 3A). Even at the lowest concentration (1 ng/mL) of STX exposure, the body length and body area were approximately 87% and 75% of the control groups, respectively (Figure 3). STX did not exhibit a dose–response relationship on the growth of worms, and no significant difference was observed among treatment groups (*p* > 0.05). 

**Figure 4:**
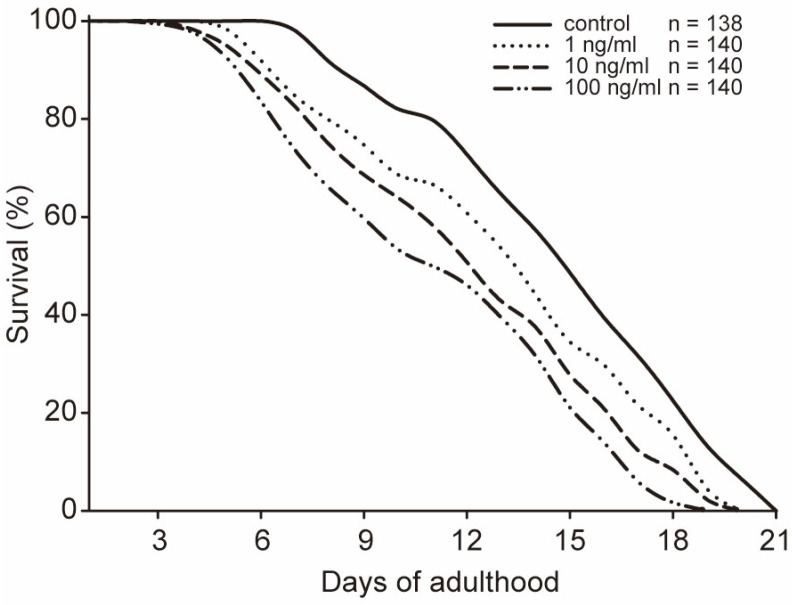
Lifespan of *C. elegans* exposed to different concentrations of STX. All data are mean values.

After exposure to STX for 48 h in 96-well plates, the worms were mainly at the young adult stage and were transferred to NGM plates to observe the lifespan. Although no worms died during the exposure experiment, the lifespan of the worms appeared to be shortened by STX. Compared with that of the controls, the mean lifespans of worms stressed by STX was 1 or 2 days shorter (Figure 4). A significant difference was observed between the 1 ng/mL and 10 ng/mL groups, although the maximum lifespans of the worms in these two groups were both 20 days. To validate the trend, we further investigated the survival time (mean ± SD) with the Kaplan–Meier survival function log-rank test. The mean survival time showed a dose-dependent trend and was 13.88 ± 0.34, 12.20 ± 0.37, 11.33 ± 0.36, and 10.38 ± 0.36 days in the control, 1, 10, and 100 ng/mL groups, respectively (Table 1). Significant differences were observed between the control and treatment groups with *p* < 0.05.

### 2.4. STX Decreases the Reproductive Function of C. elegans

**Figure 5:**
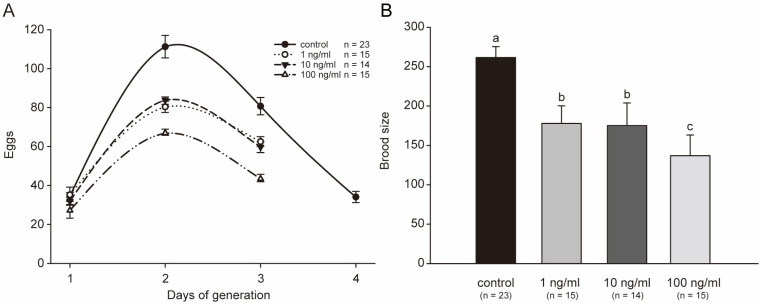
STX decreases the generation period (**A**) and the brood size (**B**). All treatments showed statistically significant differences vs. control. Bars indicate means ± SD, and different superscript letters indicate significant differences (*p* < 0.05).

Brood size usually reflects the reproductive capacity of animals. Reproductive organs may be important secondary targeted organs for toxicants in *C. elegans*. In this study, we investigated the effects of STX on the reproductive capacity of *C. elegans*. After being exposed to a series of concentrations of STX for 48 h, most worms in the treatment or control groups began to lay eggs soon after being transferred to NGM plates. The total generation trend was not perturbed by STX, and all worms laid the most eggs on the second day (Figure 5A). However, all STX treatment groups stopped breeding one day in advance. In addition, the reproductive speed (mean eggs laid each day) in the treatment group was significantly slower than that in the control group (Figure 5A). Therefore, the productivity of worms was substantially altered by STX, and the brood size was nearly 50% lower in the 100 ng/mL treatment group than in the control group (Figure 5B). No significant difference in reproductive speed was observed between 1 ng/mL and 10 ng/mL. However, the brood sizes in these two groups were approximately 67% of the control group.

### 2.5. Fluorescent Protein Expression in GFP Worms

**Figure 6:**
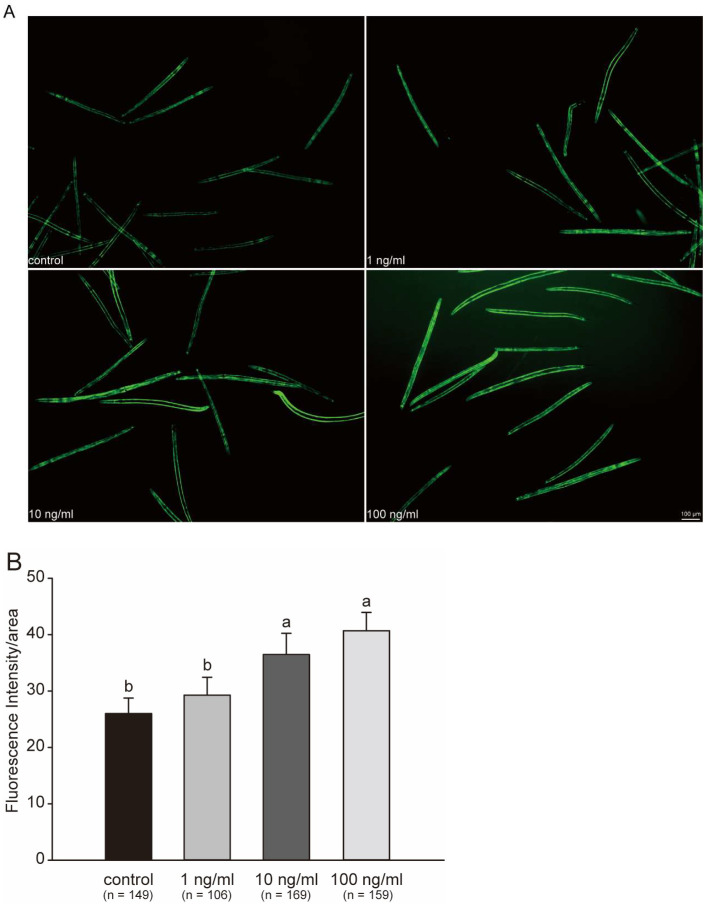
Fluorescent protein expression in GFP worms exposed to STX for 48 h (**A**) and fluorescent intensity (**B**). Intensity quantification of fluorescent protein calculated in ImageJ software (1.46r) with background removal. Bars represent means ± SD, and different superscript letters indicate significant differences (*p* < 0.05). Scale bar: 100 μm.

By testing several physiological endpoints, we observed the oxidative stress caused by STX in *C. elegans*. All these endpoints reflected potential changes in protein content. Therefore, we further performed fluorescent protein intensity quantification in the whole body in *C. elegans* to gain a general understanding of the protein expression after STX stress exposure. Figure 6A clearly indicated a difference in the fluorescence intensity. The mean fluorescence intensity in the 1, 10, and 100 ng/mL groups were 1.19, 1.62, and 2.18 times greater than that in control group, respectively, with the 10 and 100 ng/mL groups indicating a significant increase in intensity with STX concentration (Figure 6B). 

### 2.6. ATP Levels Significantly Increase after Exposure to STX 

**Figure 7:**
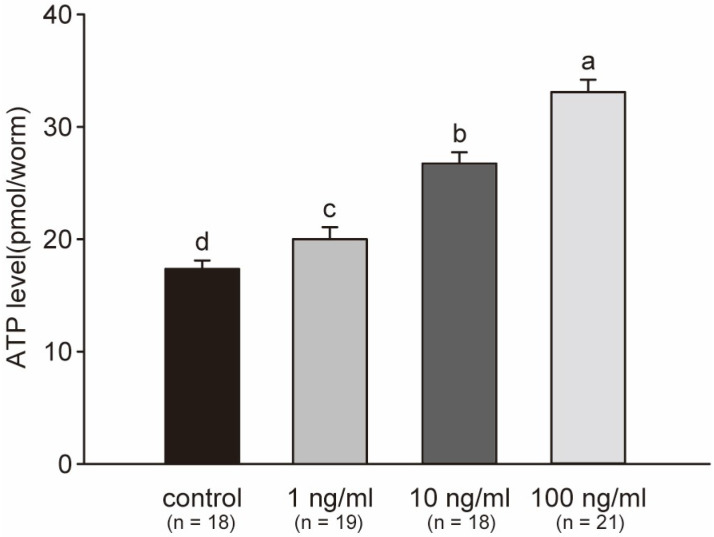
Average concentrations of ATP per worm across three independent experiments. ATP in worms significantly increased with the concentration of STX. Bars represent means ± SD, and superscript letters indicate significant differences (*p* < 0.05).

Variations in ATP levels indicate a response in cellular energy production and utilization in *C. elegans*. In this study, the ATP levels showed a significant increase with a clear dose-dependent relationship after exposure to STX for 48 h. The elevation in ATP reflected induction of energy production in *C. elegans*, because more energy was produced to defend against the stress. The average ATP level in the 1, 10 and 100 ng/mL treatment groups was 1.16, 1.54, and 1.91 times that in the control group at the end of the experiment, respectively (Figure 7). All treatment groups showed a significant difference with respect to one another.

## 3. Discussion

On the basis of the available information, PSTs are considered among the most toxic phycotoxins. According to the previous data, the mouse intraperitoneal 50% lethal dose (LD50) of STX is 10 µg/kg b.w., while the intravenous LD50 is only 3 µg/kg b.w. [1]. Compared with other toxins, for example the cobrotoxin (CBT B with an LD50 of 400 mg/kg in mice) [47], the toxicity of STX is much higher. Therefore, the occurrence of HABs associated with PST producers often leads to the mass mortality of marine organisms [14,15,23]. However, we observed that treated worms entered a state of temporary paralysis and recovered within several hours, showing high tolerance during acute exposure to STX. Gao and Zhen [43] have indicated that *C. elegans* lacks voltage-gated sodium channels. The high selective binding of Na^+^ channels is the major molecular mechanism of action of STX [26,27]. Therefore, *C. elegans* has no receptor for STX in the cell membrane, explaining the absence of neurotoxic effects of STX in *C. elegans*. On the other hand, the potassium (K^+^) channels of *C. elegans* are highly homologous to humans, controlling important behaviors, such as motility and spawning [44]. Studies have unraveled the interaction of STX with K^+^ channels. While the interaction is highly different from Na^+^ channels, it shows 100~10,000 lower binding affinities, corroborating a relatively slight toxicological impact [45]. Paralysis symptoms in *C. elegans* may be caused through K^+^ channels. 

*C. elegans* is an ideal animal model to study fitness under the stress of many environmental toxins [46,48]. Although substantial research has focused on the oxidative stress caused by STX and found that this toxin exerts adverse effects in tested animals [1], limited data are available on the chronic effects of STX in animals. In our study, even though neurotoxicity was not fatal for *C. elegans*, STX caused significant oxidative stress, which could result in chronic adverse effects. Therefore, we further investigated the responses of *C. elegans* under STX-causing oxidative stress. To validate our hypothesis, we first evaluated the changes in the lifespan of *C. elegans*. Generally, the lifespan of *C. elegans* is altered according to the stress caused by toxins, which means the aging status of this animal [49,50]. In our study, the shortened lifespan revealed an accelerated aging process for *C. elegans*, and the adult generation exposed to STX showed poorer survival than the control group. Under the stress of STX in natural seawater, it is possible that the biomass of adult organisms consistently decreases over time.

A decrease in animal lifespan indicates an intensification of aging, which may result in parental losses in the population unless more offspring are produced to compensate [51]. However, no reports have indicated that animals generate more offspring in crises; instead, parents may sacrifice themselves by decreasing their lifespan [49,51]. Many studies have found that STX producers inhibit the offspring size of marine organisms, and the parental generation experiences major damage from stress [47,52]. A decline in reproductive output caused by STX producers results primarily from the inhibition of embryonic and larval development [47,52], decreased quality of gametes [53], and decreased energy status and spermatozoa motility in marine organisms [54]. As described before, PSTs or STX cause acute toxicity by blocking voltage-gated sodium channels [26,27], thereby threatening the reproductivity of nematodes. In this study, we first indicated a decline in brood size of *C. elegans*, and observed that the offspring size was suppressed by the oxidative toxicity of STX. The generation of behavior in *C. elegans* was not significantly perturbed by STX, unlike in other animals, such as *Neomysis awatschensis* [55]. Regardless of the decline in the reproductive ability of *C. elegans*, the smaller offspring size decreased the chance of the population overcoming the adverse environment caused by the oxidative stress of STX. 

Beyond the lifespan and offspring size, biomass also depends on the body size and body weight of single organisms. Biomass directly correlates with aquaculture output. Previous studies have found that organisms might alter their body sizes under environmental stress; most tested organisms have shown a decline in body size, for example, benthic macroinvertebrates under temperature stress [56], mussels under oiling stress [57], and crabs under temperature stress [58]. In marine fish, both smaller and larger species have shown declines in size under the stresses of a century of climate change and fishing [59]. To our knowledge, only a few studies have reported the growth decline in marine organisms under the stress of PSTs and its causative dinoflagellate [10,60]. Li et al. [21] have found that *A. tamarense* depresses the growth rate of juvenile *Ruditapes philippinarum* clams, primarily because this toxic alga depresses the energy budget of clams, thus, decreasing their scope for growth. Similar results have been reported in rock lobsters fed toxic mussels contaminated with 6 mg STX.2HCL eq./kg [61].

To date, studies on the decline in biomass have focused on the energy intake depression in marine organisms under the stress of pollution, primarily the dynamic energy budget and scope for growth [62,63]. Biomass size depends not only on energy intake but also on the cellular energy status of organisms. As a result of evolution, there are strict expenditure rules, and cellular energy is distributed among three functions: basic metabolism, adaptive thermogenesis, and physical activity. [64,65]. Many studies have revealed that the energy distribution balance is perturbed, and more cellular energy is allocated to defend against stress, thus, decreasing utilization for physical activity, such as survival, growth, and reproduction [66,67,68,69]. Therefore, more research is needed to further indicate the mechanism of cellular energy distribution and to obtain a clear explanation for the marine biomass decline under the oxidative stress caused by PSTs in natural seawater. 

ROS, a commonly used indicator of oxidative stress caused by environmental stress in organisms, lead to many subsequent changes, such as damage to lipids, proteins, and DNA [67], thereby influencing physical activities including basic behaviors, and declines in lifespan, reproductive ability and body size, to adapt to the environment. Prior studies have found that additional ROS are produced in marine organisms after exposure to PSTs; toxic dinoflagellates also elicit changes in DNA/RNA damage [35,70] and antioxidant enzymes [34,35]. On the basis of our results, the ROS in *C. elegans* rose significantly during exposure, and showed clear STX dose- and body size-dependent relationships. STX enhanced the production of ROS in worms, indicating oxidative stress caused by STX. Moreover, the larger bodies of worms resulted in greater ROS production. The decline in lifespan, brood size and body size also presumably resulted from oxidative stress caused by STX, because of the absence of neurotoxicity of this toxin through binding of voltage-gated sodium channels [26,27]. 

ATP and ROS are simultaneously produced by mitochondria [71]. ATP is the basic energy currency not only for all physical activities in organisms but also for the reduction in ROS through the induction of the expression of functional proteins, such as antioxidative enzymes, to maintain the redox balance within cells and help organisms perform normal physical activities [72,73]. In our study, the total ATP level in *C. elegans* showed a dynamic rising trend during exposure, with a clearly dose-dependent relationship with STX. Additionally, ROS varieties showed a similar trend. Therefore, our results demonstrated that the increased ATP enhanced the ability of *C. elegans* to remove more ROS and thereby modulate the redox balance. The removal of ROS is implemented primarily by antioxidant enzymes, e.g., superoxide dismutase, catalase, thioredoxin, glutathione peroxidase and others [35,72,73]. The occurrence of oxidative stress or the removal of ROS both require ATP to provide sufficient energy for the antioxidant system and protect the physiological activities of organisms [74]. Under the stress of ROS, the increases in ATP also provide opportunities for organisms to adapt to environmental effects by providing more energy for reproduction, survival, or growth [67,75]. However, our study indicated that the lifespan, body size and brood size of *C. elegans* all declined under the stress of STX, while we did not analyze the potential effects of changes in antioxidant enzymes, which is one limitation of this study. As an adaptation to STX stress, these phenomena stimulate ATP allocation between survival, growth, or reproduction.

GFP is sensitive to oxidative stress, and its variation represents the oxidative damage of proteins [76]. The increasing of expression of GFP in this study indicated that stress caused protein damage in *C. elegans*. Similar results were reported by previous studies [6], and the expression of GFP in yeast has been found to significantly increase after exposure to STX. STX is believed to bind a copper transporter that is mechanistically and structurally similar to ion channels [6], the major target of STX for its neurotoxicity [26,27]. Our results contrast with those of Cusick et al. [6] in that no voltage-gated sodium channels exist in the cell membrane of *C. elegans* [43]; therefore, the protein damage in *C. elegans* was caused mainly by oxidative stress due to STX, not by the neurotoxicity through ion channel binding. At present, the mechanism through which STX induces the high expression of GFP is unclear, but protein was clearly disrupted and might have been responsible for the decline in lifespan, body size, and brood size observed in this study. In addition, the long-term presence of STX in seawater has been reported worldwide; consequently, persistent oxidative stress on marine organisms may lead to biome instability, explaining the decline in catches recent years. In the future, more research is needed to reveal the mechanisms by which marine organisms adapt to STX oxidative stress, and emphasis should be placed on cellular energy metabolism, distribution, and application.

## 4. Materials and Methods

### 4.1. Chemical and Toxin Preparation

All organic solvents used were of analytical grade and were purchased from Sigma Chemical Company (Oakville, ON, Canada) unless otherwise stated. STX standard (NRC CRM-STX-f), initially at a concentration of 65 μmol/L was purchased from the National Research Council Canada, Halifax NS, Canada. The toxin was diluted with sterile water to 10 μg/mL as a stock solution and stored at 4 °C, for use within 2 weeks. 

### 4.2. Strains 

The wild type *C. elegans* strain Bristol N2 and the GFP worm strain NL5901, as well as the OP50 *Escherichia coli* strain, were originally provided by the Caenorhabditis Genetics Center (University of Minnesota, Minneapolis, MN, USA). The worms were maintained at 20 °C on nematode growth medium (NGM) agar plates seeded with *Escherichia coli* OP50. The *C. elegans* population was treated with hypochlorite/NaOH solution to synchronize the population at day 0 of the lifespan, and larval stage 1 (L1) worms were synchronized by hatching eggs overnight. Some L1 worms were transferred to freshly spotted plates to develop into L4 worms, whereas others were directly used for treatment with STX. All treatments were performed in 96-well plates with a total volume of 100 μL containing approximately 30–40 larvae in each well. During the treatment, worms were fed with heat-inactivated bacteria (OP50 HIT) to avoid interference from the xenobiotic metabolizing activity of *E. coli* [77]. All lifespan assays were performed at 20 °C according to standard protocols, as previously described [78]. Subsequently, the worms were washed and placed on OP50-containing NGM plates to observe brood size, lifespan, and other parameters. All treatments in each experiment were performed on at least three biological replicates, and three independent experiments were performed.

### 4.3. Toxicity Assessment 

The synchronized L1 and L4 larvae stages of N2 worms were exposed to different concentrations of STX to compare the toxicity of STX on different stages of *C. elegans* development. On the basis of the prior results, the stock solution of STX was added to freshly autoclaved S-medium to a final concentration of 0, 1, 10, 50, 100, 200, 500, or 1000 ng/mL for L1 worms, and 0, 10, 100, 200, 500, 1000, 2000, 5000, or 10,000 ng/mL for L4 worms before the exposure treatment. Both treatments lasted for 24 h, and the dead worms were recorded at 0.5, 1, 2, 4, 6, 12 h, and 24 h for the L1 worms’ group and 1, 3, 6, 12, and 24 h for the L4 worms’ group. At least 100 worms were subjected to each treatment for statistical analysis. Worms’ motilities were evaluated with repeated gentle mechanical prodding, and a transient peristalsis reaction was recorded as shown paralytic symptoms. Survival was scored throughout the entire test process.

### 4.4. Measurement of ROS Production

The generation of ROS was compared between L1 and L4 larvae stages of NS worms, which were treated with 1, 10, or 100 ng/mL STX for 180 min. More than 100 animals per sample were transferred into the wells of a black 96-well plate containing 100 μL M9 buffer. Dichlorofluorescein diacetate (DCFDA; 50 mM in M9 buffer, 100 mL; Invitrogen) was added to each well. ROS-associated fluorescence levels were measured kinetically with a fluorescence plate reader (Spectra-Max Gemini EM; Molecular Devices, Sunnyvale, CA, USA) at 485 nm excitation and 520 nm emission wavelengths at room temperature, every 2 min for 3 h. Data were normalized to those for bacterial controls composed of 100 mL *E. coli* HT115 (DE3) expressing dsRNA against prdx-3 or an empty vector containing M9 buffer in separate wells of the same plate. In cells, DCFDA is deacetylated by endogenous esterases to dichlorofluorescein, which reacts with ROS and generates the fluorophore DCF. Although the specific ROS responsible for DCFDA fluorescence is uncertain, DCFDA can be used in whole *C. elegans* as a marker of general ROS production [79,80]. Three independent studies were performed. 

### 4.5. Growth Assays, Lifespan Assays and Brood Size

According to the toxicity assessment results, the synchronized L1 larvae of N2 worms were treated with 1, 10, or 100 ng/mL STX for 48 h. Worms in S-medium were used as controls.

For growth assays evaluation, worms were incubated in liquid conditions for approximately 48 h and allowed to grow to early adulthood in the absence of STX. After worms were paralyzed with tetramisole, a Nikon microscope (Eclipse E200 POL, Tokyo, Japan) equipped with a standard ruler was used to measure the body length from the posterior bulb of the pharynx to the anus, and the body area were determined by measurement of the flat surface areas of the *C. elegans* along the anterior–posterior axis in ImageJ software. At least 100 worms in each treatment were subjected to statistical analysis.

For lifespan assays evaluation, worms were transferred to fresh NGM plates with OP50 following treatment, and the time was considered day 0. On each succeeding day, worms were counted and scored as live or dead. Live *C. elegans* were picked and transferred to fresh plates every day during egg-laying and every other day after they ceased laying eggs until no live *C. elegans* remained. At least 100 worms were subjected to each treatment for statistical analysis. Survival time was determined from the survival curve data by Kaplan–Meier survival function log-rank test.

For brood size evaluation, worms were transferred to individual egg-laying plates. Animals were thereafter transferred every 24 h to fresh egg-laying plates until egg laying ceased. Eggs were kept at room temperature, and the progeny that hatched were counted after 2 days. In addition, we compared the variation in generation time from the P0 egg to the first F1 (final) egg. At least ten animals per condition per experiment were used.

### 4.6. Quantification of Fluorescent Protein Intensity in GFP Worms

To test the protein expression of *C. elegans* under the stress of STX, we incubated synchronized L1 larvae of NL5901 GFP worms in liquid conditions for 48 h in the presence of STX or S-medium (control group). After the exposure, all worms were washed three times with distilled water to remove *Escherichia coli*, then transferred to a new 96-well plate containing 50 µL S-medium. After measurement of the total fluorescence intensity under a fluorescence plate reader (Infinite 200; Tecan, Männedorf, Switzerland), we determined the protein expression with Image J software. Three independent studies were performed. 

### 4.7. Adenosine Triphosphate (ATP) Assays

Several L4 worms were randomly selected and exposed to different concentrations of STX solution for 48 h. After being washed with S-medium, the treated worms were immediately placed in liquid nitrogen for ATP measurements. The ATP assays were performed as previously described [81], with some modifications. Briefly, flash-frozen *C. elegans* were resuspended in trichloroacetic acid, lysed and incubated on ice, then subjected to centrifugation. Supernatants were pooled, and aliquots of ATP standards or sample supernatant were pipetted into white 96-well plates, and this was followed by the addition of arsenite ATP buffer. ATP levels were measured with a fluorescence plate reader (Infinite 200; Tecan, Männedorf, Switzerland). Three independent studies were performed.

### 4.8. Statistical Analyses

The statistical analyses were performed using SPSS software version27.0 (IBM Corp., Armonk, NY, USA). One-way analysis of variance (ANOVA) was used to compare body size, lifespans, brood size, GFP expression, and ATP levels among the different test groups. When significant differences were detected by ANOVA, Tukey’s honest significant difference test was performed. Statistical significance was set at *p* < 0.05.

## 5. Conclusions

PSTs and their causative dinoflagellate have been reported to reduce marine biomass by inducing oxidative stress. The presence of voltage-gated sodium channels in these organisms has complicated the study of the basic mechanisms of toxicity. This study used *C. elegans*, which lacks these channels, and found direct evidence of oxidative stress. Although our findings indicated no notable neurotoxic effects of STX in *C. elegans*, the lifespan, reproductivity, and body size of *C. elegans* all significantly declined in a dose-dependent manner during STX exposure. Therefore, STX decreases the biomass of marine organisms. Subsequently, a dynamic increase, as well as dose- and body size-dependent ROS levels in *C. elegans*, provided direct evidence of oxidative stress caused by STX. Finally, the ATP level and fluorescent protein expression both significantly increased in *C. elegans*, indicating that ATP and specific proteins might play important roles in worms or other organisms in reaction to oxidative stress caused by STX. Therefore, our study indicated the oxidative characteristics of STX on *C. elegans*. More studies are needed to clarify the mechanism of organism fitness in response to STX, particularly regarding the production, distribution, and utilization of ATP in *C. elegans* under STX stress.

## Figures and Tables

**Table 1:** Comparison of the mean lifespans for *C. elegans* exposed to STX.

Treatment	Number of *C. elegans*		Survival Time (Days)
	Total	Censored	
Control	138	0	13.88 ± 0.34 ^a^
1 ng/mL	140	0	12.20 ± 0.37 ^b^
10 ng/mL	140	0	11.33 ± 0.36 ^c^
100 ng/mL	140	0	10.38 ± 0.36 ^d^

Different superscript letters indicate significant differences (*p* < 0.05).

## Data Availability

The data presented in this study are available on request from the first author.

## References

[1] European Food Safety Authority (2009). Marine biotoxins in shellfish—Saxitoxin group. EFSA J..

[2] Landsberg J.H. (2002). The effects of harmful algal blooms on aquatic organisms. Rev. Fish. Sci..

[3] Burson A., Matthijs H., Bruijne W.D., Talens R., Huisman J. (2014). Termination of a toxic *Alexandrium* bloom with hydrogen peroxide. Harmful Algae.

[4] Cusick K.D., Minkin S.C., Dodani S.C., Chang C.J., Wilhelm S.W., Sayler G.S. (2012). Inhibition of copper uptake in yeast reveals the copper transporter Ctr1p as a potential molecular target of saxitoxin. Environ. Sci. Technol..

[5] Kozlowsky-Suzuki B., Koski M., Hallberg E., Wallen R., Carlsson P. (2009). Glutathione transferase activity and oocyte development in copepods exposed to toxic phytoplankton. Harmful Algae.

[6] Tan Z.J., Yan T., Yu R.C., Zhou M.J. (2007). Transfer of paralytic shellfish toxins via marine food chains: A simulated experiment. Biomed. Environ. Sci..

[7] Bricelj V.M., Ford S.E., Lambert C., Barbou A., Paillard C. (2011). Effects of toxic *Alexandrium tamarense* on behavior, hemocyte responses and development of brown ring disease in *Manila clams*. Mar. Ecol. Prog. Ser..

[8] Navarro J.M., Aguila B.L., Machmar F., Chaparro O.R., Contreras A.M. (2011). Dynamic of intoxication and detoxification in juveniles of *Mytilus chilensis* (Bivalvia: Mytilidae) exposed to paralytic shellfish toxins. Aquat. Living Resour..

[9] Goc N.L., Hégaret H., Fabioux C., Miner P., Soudant P. (2013). Impact of the toxic dinoflagellate *Alexandrium catenella* on Pacific oyster reproductive output: Application of flow cytometry assays on spermatozoa. Aquat. Living Resour..

[10] Nielsen M.V., Strmgren T. (1991). Shell growth response of mussels (*Mytilus edulis*) exposed to toxic microalgae. Mar. Biol..

[11] Tran D., Haberkorn H., Soudant P., Ciret P., Massabuau J.C. (2010). Behavioral responses of *Crassostrea gigas* exposed to the harmful algae *Alexandrium minutum*. Aquaculture.

[12] Yan T., Zhou M.J., Fu M., Wang L. (2003). Study on impact of dinoflagellate *Alexandrium tamarense* on life activities of marine bivalves. Acta Oceanol. Sin..

[13] Bif M.B., Yunes J.S., Resgalla C. (2013). Evaluation of mysids and sea urchins exposed to saxitoxins. Environ. Toxicol. Pharmacol..

[14] Cembella A.D., Quilliam M.A., Lewis N.I., Bauder A.G., Dell’Aversano C., Thomas K., Jellett J., Cusack R.R. (2002). The toxigenic marine dinoflagellate *Alexandrium tamarense* as the probable cause of mortality of caged salmon in Nova Scotia. Harmful Algae.

[15] Fire S.E., Pruden J., Couture D., Wang Z., Bottein M.Y.D., Haynes B.L., Knott T., Bouchard D., Lichtenwalner A., Wippelhauser G. (2012). Saxitoxin exposure in an endangered fish: Association of a shortnose sturgeon mortality event with a harmful algal bloom. Mar. Ecol. Prog. Ser..

[16] Zhang D., Hu C., Wang G., Li D., Liu Y. (2013). Zebrafish neurotoxicity from aphantoxins-cyanobacterial paralytic shellfish poisons (PSPs) from aphanizomenon flos-aquae DC-1. Environ. Toxicol..

[17] Ferrão-Filho A.D.S., Kozlowsky-Suzuki B. (2011). Cyanotoxins: Bioaccumulation and effects on aquatic animals. Mar. Drugs.

[18] Niedzwiadek B., Scott P.M., Lau P.Y. (2012). Monitoring of shrimp and farmed fish sold in Canada for cyanobacterial toxins. J. Food Prot..

[19] Jensen S.K., Lacaze J.P., Hermann G., Kershaw J., Brownlow A., Turner A., Hall A. (2015). Detection and effects of harmful algal toxins in Scottish harbour seals and potential links to population decline. Toxicon.

[20] Pearson L., Mihali T., Moffitt M., Kellmann R., Neilan B. (2010). On the chemistry, toxicology and genetics of the cyanobacterial toxins, microcystin, nodularin, saxitoxin and cylindrospermopsin. Mar. Drugs.

[21] Li S.C., Wang W.X., Hsieh D. (2002). Effects of toxic dinoflagellate *Alexandrium tamarense* on the energy budgets and growth of two marine bivalves. Mar. Environ. Res..

[22] Tian L., Cheng J., Chen X., Cheng S.H., Mak Y.L., Lam P., Chan L.L., Wang M. (2014). Early developmental toxicity of saxitoxin on medaka (*Oryzias melastigma*) embryos. Toxicon.

[23] Al-Yamani F.Y., Polikarpov I., Saburova M. (2020). Marine life mortalities and harmful algal blooms in the Northern Arabian Gulf. Aquat. Ecosyst. Health Manag..

[24] Burridge L.E., Martin J.L., Lyons M.C., Legresley M.M. (2010). Lethality of microalgae to farmed Atlantic salmon (*Salmo salar*). Aquaculture.

[25] Etheridge S.M. (2010). Paralytic shellfish poisoning: Seafood safety and human health perspectives. Toxicon.

[26] Hille B. (1975). The receptor for tetrodotoxin and saxitoxin. A structural hypothesis. Biophys. J..

[27] Shen H., Li Z., Jiang Y., Pan X., Wu J., Ben C.A., Smith J.J., Chin Y., Lei J., Zhou Q. (2018). Structural basis for the modulation of voltage-gated sodium channels by animal toxins. Science.

[28] César M., Christian L. (2014). The voltage-gated sodium channel: A major target of marine neurotoxins. Toxicon.

[29] Jiang T.J., Niu T., Xu Y.X. (2006). Transfer and metabolism of paralytic shellfish poisoning from scallop (*Chlamys nobilis*) to spiny lobster (*Panulirus stimpsoni*). Toxicon.

[30] Kwong R., Wang W.X., Lam P., Yu P. (2006). The uptake, distribution and elimination of paralytic shellfish toxins in mussels and fish exposed to toxic dinoflagellates. Aquat. Toxicol..

[31] Turner A.D., Lewis A.M., Hatfield R.G., Galloway A.W., Higman W.A. (2012). Transformation of paralytic shellfish poisoning toxins in *Crassostrea gigas* and Pecten maximus reference materials. Toxicon.

[32] Cusick K.D., Wetzel R.K., Minkin S.C., Dodani S.C., Sayler G.S. (2013). Paralytic shellfish toxins inhibit copper uptake in *Chlamydomonas reinhardtii*. Environ. Toxicol. Chem..

[33] Choi N., Yeung L., Siu W., So I., Jack R.W., Hsieh D., Wu R., Lam P. (2006). Relationships between tissue concentrations of paralytic shellfish toxins and antioxidative responses of clams, *Ruditapes philippinarum*. Mar. Pollut. Bull..

[34] Qiu J., Ma F., Hua F., Li A. (2013). Effects of feeding *Alexandrium tamarense*, a paralytic shellfish toxin producer, on antioxidant enzymes in scallops (*Patinopecten yessoensis*) and mussels (*Mytilus galloprovincialis*). Aquaculture.

[35] Fabioux C., Sulistiyani Y., Haberkorn H., Hegaret H., Amzil Z., Soudant P. (2015). Exposure to toxic *Alexandrium minutum* activates the detoxifying and antioxidant systems in gills of the oyster *Crassostrea gigas*. Harmful Algae.

[36] Gubbins M.J., Eddy F.B., Gallacher S., Stagg R.M. (2000). Paralytic shellfish poisoning toxins induce xenobiotic metabolising enzymes in Atlantic salmon (*Salmo salar*). Mar. Environ. Res..

[37] Costa P.R., Pereira P., Guilherme S., Barata M., Santos M.A., Pacheco M., Pousao-Ferreira P. (2012). Hydroxybenzoate paralytic shellfish toxins induce transient GST activity depletion and chromosomal damage in white seabream (*Diplodus sargus*). Mar. Environ. Res..

[38] Painefilú J.C., Bianchi V.A., Krock B., De Anna J.S., Kristoff G., Luquet C.M. (2020). Effects of paralytic shellfish toxins on the middle intestine of *Oncorhynchus mykiss*: Glutathione metabolism, oxidative status, lysosomal function and ATP-binding cassette class C (ABCC) proteins activity. Ecotoxicol. Environ. Saf..

[39] Durbin E., Teegarden G., Campbell R., Cembella A., Baumgartner M.F., Mate B.R. (2002). North Atlantic right whales, *Eubalaena glacialis*, exposed to paralytic shellfish poisoning (PSP) toxins via a zooplankton vector, *Calanus finmarchicus*. Harmful Algae.

[40] Doucette G.J., Cembella A.D., Martin J.L., Michaud J., Cole T., Rolland R.M. (2006). Paralytic shellfish poisoning (PSP) toxins in North Atlantic right whales *Eubalaena glacialis* and their zooplankton prey in the Bay of Fundy, Canada. Mar. Ecol. Prog..

[41] Cusick K., Gary S. (2013). An overview on the marine neurotoxin, saxitoxin: Genetics, molecular targets, methods of detection and ecological functions. Mar. Drugs.

[42] Thottumkara A.P., Parsons W.H., Du Bois J. (2014). Saxitoxin. Angew. Chem. Int. Ed..

[43] Gao S., Zhen M. (2011). Action potentials drive body wall muscle contractions in *Caenorhabditis elegans*. Proc. Natl. Acad. Sci. USA.

[44] Jiang Q., Li K., Lu W., Li S., Chen X., Liu X., Yuan J., Ding Q., Lan F., Cai S. (2018). Identification of small-molecule ion channel modulators in *C. elegans* channelopathy models. Nat. Commun..

[45] Wang J., Salata J.J., Bennett P.B. (2003). Saxitoxin is a gating modifier of HERG K^+^ channels. J. Gen. Physiol..

[46] Wang D.Y., Wang Y. (2008). Phenotypic and behavioral defects caused by barium exposure in nematode *Caenorhabditis elegans*. Arch. Environ. Contam. Toxicol..

[47] Arantes O.N., Apfeld J., Dillin A., Kenyon C. (2002). Regulation of life-span by germ-line stem cells in *Caenorhabditis elegans*. Science.

[48] Wu Y.C., Wang X., Ding X. (2012). Methods for studying programmed cell death in *C. elegans*. Methods Cell Biol..

[49] Chen X., Zhong Z., Xu Z., Chen L., Wang Y. (2010). 2′,7′-Dichlorodihydrofluorescein as a fluorescent probe for reactive oxygen species measurement: Forty years of application and controversy. Free Radic. Res. Commun..

[50] Meng Q.X., Wang W.Y., Lu Q.M., Jin Y., Wei J.F., Zhu S.W., Xiong Y.L. (2002). A novel short neurotoxin, cobrotoxin c, from monocellate cobra (*Naja kaouthia*) venom: Isolation and purification, primary and secondary structure determination, and tertiary structure modeling. Comp. Biochem. Physiol. Part C Toxicol. Pharmacol..

[51] Zhang Y., Zhao C., Zhang H., Liu R., Yin L. (2021). Integrating transcriptomics and behavior tests reveals how the *C. elegans* responds to copper induced aging. Ecotoxicol. Environ. Saf..

[52] Ros-Santaella J.L. (2021). Impact of oxidative stress on male reproduction in domestic and wild animals. Antioxidants.

[53] Tian Y., Zhou M., Meng F., Yu R., Wang Y., Li J. (2003). Effects of the dinoflagellate *Alexandrium tamarense* on early development of the scallop *Argopecten irradians* concentricus. Aquaculture.

[54] Yan T., Zhou M., Fu M., Wang Y., Yu R., Li J. (2001). Inhibition of egg hatching success and larvae survival of the scallop, *Chlamys farreri*, associated with exposure to cells and cell fragments of the dinoflagellate *Alexandrium tamarense*. Toxicon.

[55] Yurchenko O.V., Radashevsky V.I., Hsieh H.L., Reunov A.A. (2009). Ultrastructural comparison of the spermatozoa of the Pacific oyster *Crassostrea gigas* inhabiting polluted and relatively clean areas in Taiwan. Aquat. Ecol..

[56] Haberkorn H., Lambert C., Goiec N.L., Moal J., Suquet M., Gueguen M., Sunila I., Soudant P. (2010). Effects of *Alexandrium minutum* exposure on nutrition-related processes and reproductive output in oysters *Crassostrea gigas*. Harmful Algae.

[57] Zhijun T., Tian Y., Mingjiang Z., Jun L., Rencheng Y., Yunfeng W. (2002). The effects of *Alexandrium tamarense* on survival, growth and reproduction of *Neomysis awatschensis*. Acta Ecol. Sin..

[58] Piazza V., Ullmann C.V., Aberhan M. (2020). Temperature-related body size change of marine benthic macroinvertebrates across the Early Toarcian Anoxic Event. Sci. Rep..

[59] Turner R.E., Plunket J.S. (2021). Estuarine oiling increases a long-term decline in mussel growth. Environ. Pollut..

[60] Grande F.R.D., Granado P., Costa T.M. (2021). Size-at-age or structure shift: Which hypothesis explains smaller body size of the fiddler crab Leptuca uruguayensis in northern populations?. Estuar. Coast. Shelf Sci..

[61] Genner M.J., Sims D.W., Southward A.J., Budd G.C., Masterson P., Mchugh M., Rendle P., Southall E.J., Wearmouth V.J., Hawkins S.J. (2010). Body size-dependent responses of a marine fish assemblage to climate change and fishing over a century-long scale. Glob. Chang. Biol..

[62] Luckenbach M.W., Sellner K.G., Shumway S.E., Greene K. (1993). Effects of two bloom-forming dinoflagellates, *Prorocentrum* mariae-lebouriae and *Gyrodinium unctenumon* on the growth and survival of the eastern oyster, *Crassostrea virginica* (Gmelin, 1791). J. Shellfish Res..

[63] Turnbull A., Malhi N., Seger A., Jolley J., Fitzgibbon Q. (2020). Accumulation of paralytic shellfish toxins by Southern Rock Lobster *Jasus edwardsii* causes minimal impact on lobster health. Aquat. Toxicol..

[64] Duan J., Liu H., Zhu J., Lu L., Chang L. (2021). A dynamic energy budget model for abalone, *Haliotis discus hannai* Ino. Ecol. Model..

[65] Pousse E., Flye-Sainte-Marie J., Alunno-Bruscia M., Hegaret H., Rannou E., Pecquerie L., Marques G.M., Thomas Y., Castrec J., Fabioux C. (2019). Modelling paralytic shellfish toxins (PST) accumulation in *Crassostrea gigas* by using Dynamic Energy Budgets (DEB). J. Sea Res..

[66] Bruynsteen L., Janssens G.P.J., Harris P.A., Duchateau L., Valle E., Odetti P., Vandevelde K., Buyse J., Hesta M. (2014). Changes in oxidative stress in response to different levels of energy restriction in obese ponies. Br. J. Nutr..

[67] Rolfe D.F., Brown G.C. (1997). Cellular energy utilization and molecular origin of standard metabolic rate in mammals. Physiol. Rev..

[68] Stenesen D., Suh J.M., Seo J., Yu K., Lee K.S., Kim J.S., Min K.J., Graff J.M. (2013). Adenosine nucleotide biosynthesis and AMPK regulate adult life span and mediate the longevity benefit of caloric restriction in flies. Cell Metab..

[69] Ranjan M., Gruber J., Ng L.F., Halliwell B. (2013). Repression of the mitochondrial peroxiredoxin antioxidant system does not shorten life span but causes reduced fitness in *Caenorhabditis elegans*. Free Radic. Biol. Med..

[70] Wu S.B., Wei Y.H. (2012). AMPK-mediated increase of glycolysis as an adaptive response to oxidative stress in human cells: Implication of the cell survival in mitochondrial diseases. Biochim. Biophys. Acta-Mol. Basis Dis..

[71] Yanase S., Yasuda K., Ishii N. (2002). Adaptive responses to oxidative damage in three mutants of *Caenorhabditis elegans* (age-1, mev-1 and daf-16) that affect life span. Mech. Ageing Dev..

[72] Acba B., Vp B., Rm B., Am B., Sg B., Prca C., Mp B. (2020). DNA damage and oxidative stress responses of mussels *Mytilus galloprovincialis* to paralytic shellfish toxins under warming and acidification conditions—Elucidation on the organ-specificity. Aquat. Toxicol..

[73] Almansa-Ordonez A., Bellido R., Vassena R., Barragan M., Zambelli F. (2020). Oxidative stress in reproduction: A mitochondrial perspective. Biology.

[74] Napolitano G., Fasciolo G., Venditti P. (2021). Mitochondrial management of reactive oxygen species. Antioxidants.

[75] Wang Y., Sun Y., Zhang Z., Li Z., Cai P. (2020). Enhancement in the ATP level and antioxidant capacity of *Caenorhabditis elegans* under continuous exposure to extremely low-frequency electromagnetic field for multiple generations. Int. J. Radiat. Biol..

[76] Kanaan G.N., Harper M.E. (2017). Cellular redox dysfunction in the development of cardiovascular diseases. Biochim. Biophys Acta Gen. Subj..

[77] Zhang J., Shi R., Li H., Xiang Y., Xiao L., Hu M., Ma F., Ma C.W., Huang Z. (2016). Antioxidant and neuroprotective effects of Dictyophora indusiata polysaccharide in *Caenorhabditis elegans*. J. Ethnopharmacol..

[78] Wen H., Shi W., Qin J. (2012). Multiparameter evaluation of the longevity in *C. elegans* under stress using an integrated microfluidic device. Biomed. Microdevices.

[79] *C. elegans* Sequencing Consortium (1998). Genome Sequence of the Nematode *C. elegans*: A Platform for Investigating Biology. Science.

[80] Gruber J., Ng L.F., Poovathingal S.K., Halliwell B. (2009). Deceptively simple but simply deceptive—*Caenorhabditis elegans* lifespan studies: Considerations for aging and antioxidant effects. FEBS Lett..

[81] Schulz T.J., Zarse K., Voigt A., Urban N., Birringer M., Ristow M. (2007). Glucose restriction extends *Caenorhabditis elegans* life span by inducing mitochondrial respiration and increasing oxidative stress. Cell Metab..

